# A population-based predictive model to identify patients with signet ring cell carcinoma of the stomach who are most suitable for primary tumor resection

**DOI:** 10.1186/s12957-022-02544-y

**Published:** 2022-03-16

**Authors:** Biao Hu, Run-Pu Zou, Yin-Wen Gan, Yi-Hao Zhu, Si-Min Ren, Wei-Zhong Hou, Zhi-Xin Xie, Ru Wang, Wen-Ting Yang, Peng-Ji Lin, Jun-Tao Feng, Zi-Min Gao, Xu-Guang Guo

**Affiliations:** 1 Department of Clinical Laboratory Medicine, The Third Affiliated Hospital of Guangzhou Medical University, Guangzhou, 510150 China; 2 Department of Clinical Medicine, The Second Clinical School of Guangzhou Medical University, Guangzhou, 511436 China; 3 Department of Medical Imaging, The Second Clinical School of Guangzhou Medical University, Guangzhou, 511436 China; 4 Department of Clinical Medicine, The Third Clinical School of Guangzhou Medical University, Guangzhou, 511436 China; 5 Key Laboratory for Major Obstetric Diseases of Guangdong Province, The Third Affiliated Hospital of Guangzhou Medical University, Guangzhou, 510150 China; 6 Key Laboratory of Reproduction and Genetics of Guangdong Higher Education Institutes, The Third Affiliated Hospital of Guangzhou Medical University, Guangzhou, 510150 China

**Keywords:** Signet ring carcinoma of the stomach, Predictive model, Surgery, SEER database, Nomogram

## Abstract

**Background:**

Though the survival benefit of primary tumor operation for patients with signet ring cell carcinoma of the stomach is known, the specific characteristics of those patients who would profit from the operation are yet to be determined. To this end, a predictive model was developed to identify the conjecture that the survival profit from primary tumor operation would only be obtained by patients.

**Method:**

The clinical data of the patients with signet ring cell carcinoma of the stomach were obtained from the Surveillance, Epidemiology, and End Results database, and then divided into operation and no-operation groups based on whether the patients underwent the primary tumor operation. To remove the confounding factors, propensity score matching was employed, and it was hypothesized that the patients who had been operated on and lived a longer life than the median cancer-specific survival time of those who hadn’t must have profited from the surgery. To discuss the independent factors of cancer-specific survival time in the beneficial group and the non-beneficial group, the Cox model was used, and based on the various vital predictive factors, a nomogram was drawn using logistic regression.

**Result:**

The number of eligible patients was 12,484, with 43.9% (5483) of them having received surgery. After employing propensity score matching, the cancer-specific survival time of the operation group was found to be apparently longer (median: 21 vs. 5 months; *p* < 0.001) than the no-operation group. In the operation group, 4757 (86.7%) of the patients lived longer than five months (beneficial group). The six indexes (beneficial and non-beneficial group) included gender, age, Tumor Node Metastasis stage, histologic type, differentiation grade, and tumor position, and were used as predictors to draw the nomogram. The nomogram was used to divide the patients who had taken operations into two groups: the beneficial operation group and the non-beneficial operation group. The beneficial operation group, it was found, survived longer than the non-beneficial operation group (median cancer-specific survival time: 28 vs. 3 months, *p* < 0.001). Moreover, there was we could tell little difference in survival between the two groups (median cancer-specific survival time: 3 vs. 5 months).

**Conclusions:**

The predictive model created to select suitable candidates for surgical treatment from patients with signet ring carcinoma of the stomach could be adopted to identify certain patients benefiting from the primary tumor operation.

## Introduction

Gastric cancer is the fifth most commonplace cancer worldwide and the third leading cause of cancer-related death [1, 2]. Although there has been a decrease in the cumulative incidence of gastric cancer in recent years, the incidence of signet ring cell carcinoma (SRCC), recent studies show, has been ceaselessly rising, accounting for 35–45% of the gastric adenocarcinoma cases in Europe, Asia, and the USA [3, 4].

Lauren, Ming, and Nakamura separately defined SRCC as “diffuse type,” “infiltrative type,” and “undifferentiated type,” respectively. Nowadays, it is defined as a poorly cohesive carcinoma, with a cell rich in intracytoplasmic mucin pushing the nucleus to the periphery [5–7].

For patients with SRCC of the stomach, recent studies suggest that the preferable approach is resection. However, not all patients can profit from this operation [8]. The prognosis, for example, is different between early gastric cancer and advanced gastric cancer, which may probably be related to the fact that SRCC in early gastric cancer and advanced gastric cancer may represent two distinct subsets.

The potential benefit of primary tumor surgery in SRCC might differ among patients because of their unique characteristics, but there is no complete clarity on these characteristics. To fill this gap, this study developed a predictive model using a prospective national database to determine that the patients will obtain a survival profit from the primary tumor operation and identify, from among patients with SRCC of the stomach, the proper candidates for primary tumor operation [2].

## Method

### Using SEER database to select patients

The Surveillance, Epidemiology, and End Results (SEER) database is a national population-based reporting system that regularly collects clinical retrospective data such as patient demographics, primary tumor location, tumor morphology, partial immunohistochemistry, diagnostic stage, the first course of treatment, and survival status follow-up. But there are no identifiers in the SEER data that can be disclosed for cancer-based epidemiological research and survival analysis. We obtained access to the data (SEER stat user name: 14406-nov2020) and, based on website code classification, extracted the gastric cancer cases diagnosed from 2000 to 2018 from the database (SEER stat 8.3.9). We chose this period because we wanted to ensure a long-enough follow-up time and because we had the data on Tumor Node Metastasis (TNM) staging and collaboration period (CS) from the American Joint Commission on Cancer (AJCC) from patients diagnosed between 2000 and 2018.

After patient selection, their clinical and tumor characteristics and their survival outcome data, including cancer-specific survival (CSS) and overall survival (OS), were recorded. After reclassifying the TNM stage based on the sixth edition of AJCC, patients with histologically confirmed gastric cancer were also be included. If the surgical record of the primary site was unknown, the patient was excluded. The other criteria for exclusion included unknown operation code, unknown TNM stage, unknown survival months, unknown race, and unknown cause of death.

### Ethics statement

The study conformed with the Declaration of Helsinki (as revised in 2013), which establishes the ethical principles for medical research involving human subjects and is an ethical principle and restrictive condition for biomedical research including human subjects. Identifiable human material and data were studied, and the data extracted from SEER was made public and de-identified.

### Statistical analysis

Based on whether surgery was performed, the samples were categorized into two groups: the surgical group and the non-surgical group. Age, race, degree of differentiation, blood therapy, marital status, radiotherapy, and other variables that may affect treatment results were analyzed by logistic regression to generate the propensity score. Propensity score matching (PSM) was used since it can decrease the effect of data error and confounding variables and can help reasonably match the two groups of patients (R software version 4.0.5, https://www.rproject.org/). The two groups were matched with the closest propensity score on the logit scale and used the closest propensity score on the logarithmic scale to match the corrector 0.1.

The overall survival (OS) and cancer-specific survival (CSS) of patients with gastric cancer were obtained from the SEER database. OS refers to the time from random to death diagnosed from any cause, while CSS calculates the time from the date of diagnosis to the cancer-specific date of death. To determine the independent prognostic factors related to OS and CSS in SRCC patients, the multivariate Cox proportional hazards regression analysis was used. To estimate OS and CSS, the Kaplan-Meier (K-M) method was used. The hazard ratio (HR) was calculated using a 95% confidence interval (CI). The SPSS 24.0 software (IBM Corporation, Armonk, NY, USA) and the R version 4.0.5 software were used (http://www.r-project.org/) to perform statistical analysis.

### Crafting a nomogram

#### Construction

We assumed that the patients who underwent the resection of primary tumors had a longer median CSS survival time (5 months, the result of PSM post-processing data) than those without. Based on this, we divided the groups into two: the beneficial group and the non-beneficial group. The eligible patients who underwent primary tumor resection were stochastically divided into training set and validation set at 7:3. The multivariate Cox analysis was then used, and the independent influence variables obtained before the operation—age, race, summary stage (distant), chemistry and systematic surgery and surgery to the primary site, and other CSS-affecting factors—are included in the training set (see Table 2 for details). Based on the multivariate logistic analysis, we established the nomogram gastric signet ring cell carcinoma prediction model using R software (version 4.0.5) and RMS package to predict the patients in the training set who can really profit from the initial operation.

### Verify nomogram

#### Validation

The data of the survival analysis and nomogram are from the training set, and the data of the validation prediction model are from the validation set. First, the mammogram’s performance was quantified in training, followed by the verification of its identification, calibration, and clinical utility. Based on the validation set, the area under the patient operating characteristic (ROC) curve (AUC) and the calibration map were obtained using regression analysis. Then, the identification ability and accuracy of the nomogram were evaluated respectively. AUC ranged from 0.5 to 1. The larger the value, the higher the prediction accuracy. In our prediction model, we divided the gastric cancer patients after PSM from the SEER database into three groups: the non-surgical group, the surgical benefit group, and the surgical non-benefit group. To this end, we established a standard to determine whether the candidates really benefited from the resection of primary tumors: the patients with a benefit probability > 0.5 were classified as candidates in the benefit group, while the patients with a benefit probability ≤ 0.5 were classified as candidates in the non-beneficial group. Then, using the Kaplan-Meier analysis, we examined whether the model could distinguish the patients who can profit from primary tumor resection.

## Results

### Patient characteristics

**Fig. 1:**
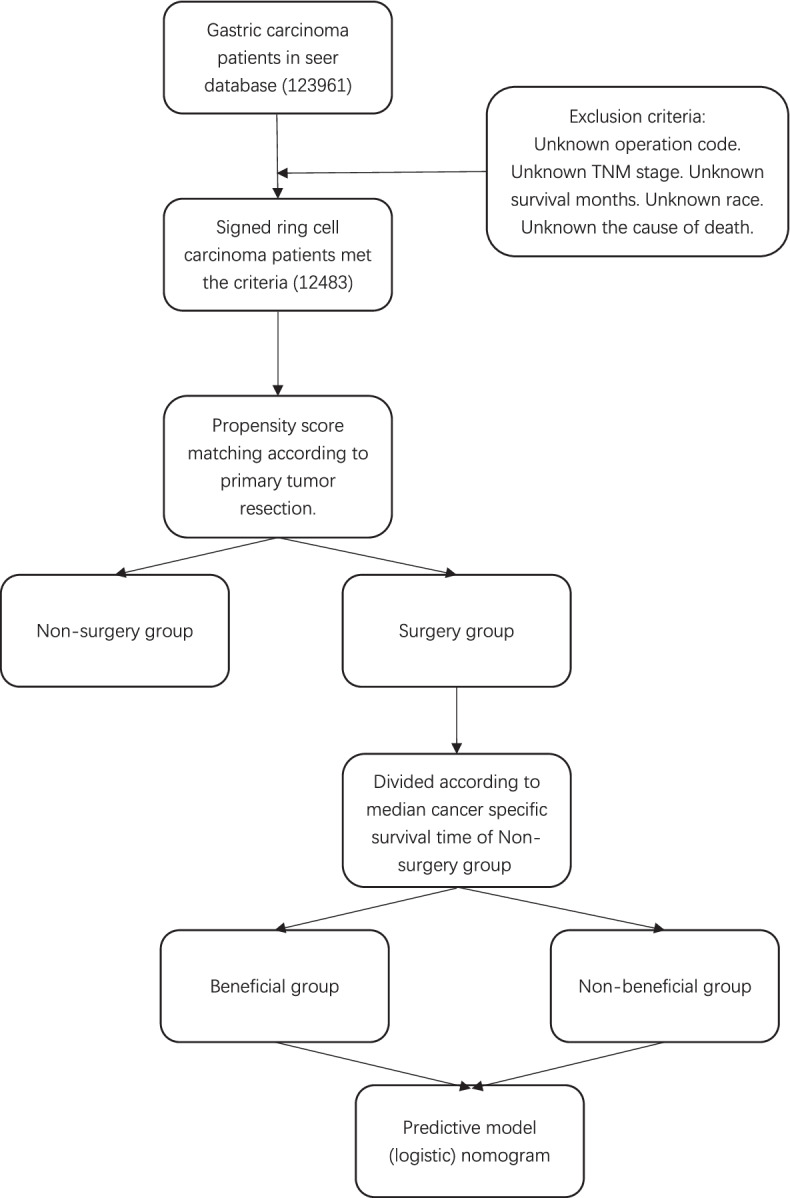
Flow chart of predictive model construction. SEER, Surveillance, Epidemiology, and End Results; PSM, propensity score matching

Our study cohort included a total of 123,961 gastric carcinoma (GC) patients between 2000 and 2018 obtained from the SEER database. The data on a total of 12484 patients with SRCC were extracted according to the screening criteria (Fig. 1). Out of the eligible patients, the samples were divided into two groups, surgery and non-surgery groups, based on whether the resection of the primary tumor site was performed, of which 5483 (43.92%) patients received surgical treatment. The groups had significant differences in age, race, gender, primary tumor site, summary stage, TNM stage, chemotherapy, systematic treatment, metastatic sites, resection range, diagnosis time, and marital status (*p* < 0.01 (Table 1). There was also an imbalance in the baseline characteristics of the two groups. Therefore, we used PSM to create well-balanced groups.

**Table 1:** Demographic information for patients with signet ring cell carcinoma before PSM

	All patients (*n* = 12483), *n* (%)	Non-surgery group (*n* = 6996), *n* (%)	Surgery group (*n* = 5487), *n* (%)	*P*
Age				< 0.001
< 50	2457 (19.7)	1373 (19.6)	1084 (19.8)	
50–59	2561 (20.5)	1344 (19.2)	1217 (22.2)	
60–69	2916 (23.4)	1528 (21.8)	1388 (25.3)	
70–79	2609 (20.9)	1389 (19.9)	1220 (22.2)	
> 80	1940 (15.5)	1362 (19.5)	578 (10.5)	
Gender				0.312
Female	5989 (48.0)	3328 (47.6)	2661 (48.5)	
Male	6494 (52.0)	3668 (52.4)	2826 (51.5)	
Race				
Black	1569 (12.6)	898 (12.8)	671 (12.2)	
White	8933 (71.6)	5154 (73.7)	3779 (68.9)	
Other	1981 (15.9)	944 (13.5)	1037 (18.9)	
**Primary site**				< 0.001
Cardia, NOS	2207 (17.7)	1417 (20.3)	790 (14.4)	
Fundus of stomach	386 ( 3.1)	251 (3.6)	135 (2.5)	
Body of stomach	1487 (11.9)	894 (12.8)	593 (10.8)	
Gastric antrum	2688 (21.5)	1180 (16.9)	1508 (27.5)	
Pylorus	364 ( 2.9)	131 (1.9)	233 (4.2)	
Lesser curvature of stomach, NOS	1022 ( 8.2)	362 (5.2)	660 (12)	
Greater curvature of stomach, NOS	526 ( 4.2)	228 (3.3)	298 (5.4)	
Overlapping lesion of stomach	1332 (10.7)	745 (10.6)	587 (10.7)	
Stomach, NOS	2471 (19.8)	1788 (25.6)	683 (12.4)	
**Summary stage**				< 0.001
Unknown/unstaged	968 ( 7.8)	906 (13)	62 (1.1)	
Distant	5267 (42.2)	4330 (61.9)	937 (17.1)	
Localized	2313 (18.5)	924 (13.2)	1389 (25.3)	
Regional	3935 (31.5)	836 (11.9)	3099 (56.5)	
**AJCC stage group**				< 0.001
Unknown stage	1344 (10.8)	1212 (17.3)	132 (2.4)	
IA	1457 (11.7)	590 (8.4)	867 (15.8)	
IB	1059 ( 8.5)	317 (4.5)	742 (13.5)	
II	1280 (10.3)	268 (3.8)	1012 (18.4)	
IIIA	1149 ( 9.2)	276 (3.9)	873 (15.9)	
IIIB	360 ( 2.9)	10 (0.1)	350 (6.4)	
IV	5834 (46.7)	4323 (61.8)	1511 (27.5)	
**T stage**				< 0.001
T0	30 ( 0.2)	29 (0.4)	1 (0)	
T1	2511 (20.1)	1414 (20.2)	1097 (20)	
T2a	858 ( 6.9)	396 (5.7)	462 (8.4)	
T2b	2320 (18.6)	717 (10.2)	1603 (29.2)	
T2NOS	94 ( 0.8)	60 (0.9)	34 (0.6)	
T3	1942 (15.6)	394 (5.6)	1548 (28.2)	
T4	1800 (14.4)	1173 (16.8)	627 (11.4)	
TX	2928 (23.5)	2813 (40.2)	115 (2.1)	
**N stage**				< 0.001
N0	5112 (41.0)	3191 (45.6)	1921 (35)	
N1	3436 (27.5)	1642 (23.5)	1794 (32.7)	
N2	1207 ( 9.7)	120 (1.7)	1087 (19.8)	
N3	641 ( 5.1)	72 (1)	569 (10.4)	
NX	2087 (16.7)	1971 (28.2)	116 (2.1)	
**M stage**				< 0.001
M0	6689 (53.6)	2146 (30.7)	4543 (82.8)	
M1	5022 (40.2)	4174 (59.7)	848 (15.5)	
MX	772 ( 6.2)	676 (9.7)	96 (1.7)	
**Radiation**				< 0.001
No	9561 (76.6)	5946 (85)	3615 (65.9)	
Yes	2922 (23.4)	1050 (15)	1872 (34.1)	
**Chemotherapy**				< 0.001
No	5922 (47.4)	3493 (49.9)	2429 (44.3)	
Yes	6561 (52.6)	3503 (50.1)	3058 (55.7)	
**Systemic surgery**				< 0.001
No	9758 (78.2)	6659 (95.2)	3099 (56.5)	
Yes	2725 (21.8)	337 (4.8)	2388 (43.5)	
**DX bone**				< 0.001
No	5457 (43.7)	3015 (43.1)	2442 (44.5)	
Yes	413 ( 3.3)	398 (5.7)	15 (0.3)	
Unknown	6613 (53.0)	3583 (51.2)	3030 (55.2)	
**DX liver**				< 0.001
No	5495 (44.0)	3056 (43.7)	2439 (44.5)	
Yes	362 ( 2.9)	338 (4.8)	24 (0.4)	
Unknown	6626 (53.1)	3602 (51.5)	3024 (55.1)	
**DX lung**				< 0.001
No	5576 (44.7)	3131 (44.8)	2445 (44.6)	
Yes	270 ( 2.2)	254 (3.6)	16 (0.3)	
Unknown	6637 (53.2)	3611 (51.6)	3026 (55.1)	
**Resection range**				< 0.001
No	6996 (56.0)	6996 (100)	0 (0)	
Local tumor resection	114 ( 0.9)	0 (0)	114 (2.1)	
Not-total gastrectomy surgery	3436 (27.5)	0 (0)	3436 (62.6)	
Total gastrectomy surgery	1824 (14.6)	0 (0)	1824 (33.2)	
Unknown	113 ( 0.9)	0 (0)	113 (2.1)	
**Year of diagnosis**				< 0.001
2004–2007	4188 (33.5)	2125 (30.4)	2063 (37.6)	
2008–2011	4075 (32.6)	2265 (32.4)	1810 (33)	
2012–2015	4220 (33.8)	2606 (37.2)	1614 (29.4)	
**Marital**				< 0.001
No	2667 (21.4)	1651 (23.6)	1016 (18.5)	
Yes	9816 (78.6)	5345 (76.4)	4471 (81.5)	

After the 1:1 PSM, the survival analysis included 3002 SRCC patients who did or did not undergo primary tumor resection. And in the post-PSM data, the baseline characteristics—age, race, gender, primary tumor site, summary stage, TNM stage, chemotherapy, systematic treatment, diagnosis time, metastatic sites, resection range, and marital status—were all balanced (*p* > 0.05) (Table 2).

**Table 2:** Demographic information for patients with signet ring cell carcinoma after PSM

	All patients (*n* = 3002), *n* (%)	Non-surgery group (*n* = 1501), *n* (%)	Surgery group (*n* = 1501), n (%)	*P*
**Age**				0.684
< 50	525 (17.5)	266 (17.7)	259 (17.3)	
50–59	553 (18.4)	278 (18.5)	275 (18.3)	
60–69	631 (21.0)	320 (21.3)	311 (20.7)	
70–79	692 (23.1)	329 (21.9)	363 (24.2)	
> 80	601 (20.0)	308 (20.5)	293 (19.5)	
**Gender**				1
Female	1476 (49.2)	738 (49.2)	738 (49.2)	
Male	1526 (50.8)	763 (50.8)	763 (50.8)	
**Race**				0.214
Black	365 (12.2)	194 (12.9)	171 (11.4)	
White	2153 (71.7)	1079 (71.9)	1074 (71.6)	
Other	484 (16.1)	228 (15.2)	256 (17.1)	
**Primary site**				0.959
Cardia, NOS	562 (18.7)	271 (18.1)	291 (19.4)	
Fundus of stomach	99 ( 3.3)	50 (3.3)	49 (3.3)	
Body of stomach	355 (11.8)	176 (11.7)	179 (11.9)	
Gastric antrum	726 (24.2)	361 (24.1)	365 (24.3)	
Pylorus	80 ( 2.7)	43 (2.9)	37 (2.5)	
Lesser curvature of stomach, NOS	204 ( 6.8)	110 (7.3)	94 (6.3)	
Greater curvature of stomach, NOS	124 ( 4.1)	61 (4.1)	63 (4.2)	
Overlapping lesion of stomach	300 (10.0)	151 (10.1)	149 (9.9)	
Stomach, NOS	552 (18.4)	278 (18.5)	274 (18.3)	
**Summary stage**				0.684
Unknown/unstaged	107 ( 3.6)	49 (3.3)	58 (3.9)	
Distant	951 (31.7)	481 (32)	470 (31.3)	
Localized	1060 (35.3)	538 (35.8)	522 (34.8)	
Regional	884 (29.4)	433 (28.8)	451 (30)	
**AJCC stage group**				0.915
Unknown Stage	199 ( 6.6)	95 (6.3)	104 (6.9)	
IA	800 (26.6)	410 (27.3)	390 (26)	
IB	352 (11.7)	174 (11.6)	178 (11.9)	
II	330 (11.0)	168 (11.2)	162 (10.8)	
IIIA	241 ( 8.0)	113 (7.5)	128 (8.5)	
IIIB	19 ( 0.6)	10 (0.7)	9 (0.6)	
IV	1061 (35.3)	531 (35.4)	530 (35.3)	
**T stage**				0.978
T0	1 ( 0.0)	0 (0)	1 (0.1)	
T1	1000 (33.3)	514 (34.2)	486 (32.4)	
T2a	236 ( 7.9)	118 (7.9)	118 (7.9)	
T2b	580 (19.3)	288 (19.2)	292 (19.5)	
T2NOS	18 ( 0.6)	9 (0.6)	9 (0.6)	
T3	421 (14.0)	207 (13.8)	214 (14.3)	
T4	539 (18.0)	264 (17.6)	275 (18.3)	
TX	207 ( 6.9)	101 (6.7)	106 (7.1)	
**N stage**				0.263
N0	1555 (51.8)	794 (52.9)	761 (50.7)	
N1	952 (31.7)	471 (31.4)	481 (32)	
N2	173 ( 5.8)	78 (5.2)	95 (6.3)	
N3	93 ( 3.1)	39 (2.6)	54 (3.6)	
NX	229 ( 7.6)	119 (7.9)	110 (7.3)	
**M stage**				0.75
M0	1998 (66.6)	990 (66)	1008 (67.2)	
M1	893 (29.7)	456 (30.4)	437 (29.1)	
MX	111 ( 3.7)	55 (3.7)	56 (3.7)	
**Radiation**				0.279
No	2448 (81.5)	1236 (82.3)	1212 (80.7)	
Yes	554 (18.5)	265 (17.7)	289 (19.3)	
**Chemotherapy**				0.679
No	1870 (62.3)	941 (62.7)	929 (61.9)	
Yes	1132 (37.7)	560 (37.3)	572 (38.1)	
**Systemic surgery**				0.958
No	2576 (85.8)	1289 (85.9)	1287 (85.7)	
Yes	426 (14.2)	212 (14.1)	214 (14.3)	
**Diagnosis**				0.26
2004–2007	1242 (41.4)	600 (40)	642 (42.8)	
2008–2011	874 (29.1)	442 (29.4)	432 (28.8)	
2012–2015	886 (29.5)	459 (30.6)	427 (28.4)	
**Marital**				0.686
No	626 (20.9)	318 (21.2)	308 (20.5)	
Yes	2376 (79.1)	1183 (78.8)	1193 (79.5)	
**DX bone**				< 0.001
No	1236 (41.2)	639 (42.6)	597 (39.8)	
Yes	32 ( 1.1)	28 (1.9)	4 (0.3)	
Unknown	1734 (57.8)	834 (55.6)	900 (60)	
**DX liver**				0.003
No	1235 (41.1)	640 (42.6)	595 (39.6)	
Yes	34 ( 1.1)	25 (1.7)	9 (0.6)	
Unknown	1733 (57.7)	836 (55.7)	897 (59.8)	
**DX lung**				0.024
No	1240 (41.3)	645 (43)	595 (39.6)	
Yes	30 ( 1.0)	20 (1.3)	10 (0.7)	
Unknown	1732 (57.7)	836 (55.7)	896 (59.7)	
**Resection range**				< 0.001
No	1501 (50.0)	1501 (100)	0 (0)	
Local tumor resection	84 ( 2.8)	0 (0)	84 (5.6)	
Not-total gastrectomy surgery	921 (30.7)	0 (0)	921 (61.4)	
Total gastrectomy surgery	427 (14.2)	0 (0)	427 (28.4)	
Unknown	69 ( 2.3)	0 (0)	69 (4.6)	

### Effect of primary tumor surgery on the survival outcomes of SRCC patients

**Fig. 2:**
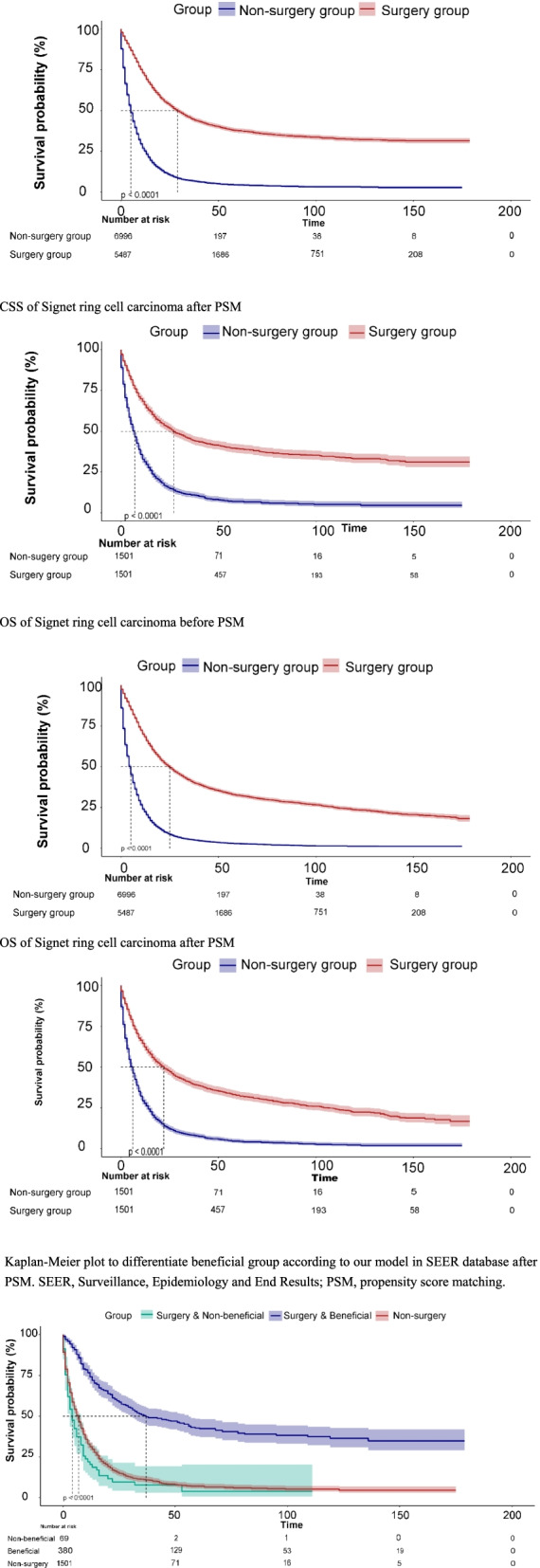
Kaplan-Meier plot of signet ring cell carcinoma patients according to treatment. SEER, Surveillance, Epidemiology, and End Results; PSM, propensity score matching; CSS, cancer-specific survival; OS, overall survival. **a** CSS of signet ring cell carcinoma before PSM. **b** CSS of signet ring cell carcinoma after PSM. **c** OS of signet ring cell carcinoma before PSM. **d** OS of signet ring cell carcinoma after PSM. **e** Kaplan-Meier plot to differentiate beneficial group according to our model in SEER database after PSM. SEER, Surveillance, Epidemiology, and End Results; PSM, propensity score matching

According to the results (Table 3, Fig. 2), in the K-M analysis and log-rank test, the surgery group had longer OS and CSS in the matched population. The median CSS of patients who underwent primary tumor resection was 21 months compared to the 5 months in the non-surgical group after PSM (HR = 36%, 95% CI, 0.34–0.40, *p* < 0.001). In the surgery group, the 1-year CSS rate was 66.1%, and the 3-year was 44.9%. In the non-surgery group, the 1-year CSS rate was 31.4%, and the 3-year was 11.1% (Table 4).

**Table 3:** Multivariate Cox analysis for OS and CSS among PSM population

	CSS		OS	
	Adjust HR (95% CI)	*p* value	Adjust HR (95% CI)	*p* value
**Age**				
< 50	Reference			
50–59	1.09 (0.94~1.25)	0.247	1.13 (0.99~1.3)	0.072
60–69	1.21 (1.05~1.39)	0.007	1.29 (1.13~1.48)	< 0.001
70–79	1.35 (1.16~1.55)	< 0.001	1.54 (1.35~1.77)	< 0.001
> 80	1.81 (1.55~2.11)	< 0.001	2.17 (1.87~2.5)	< 0.001
**Gender**				
Female	Reference			
Male	1.06 (0.97~1.16)	0.203	1.06 (0.97~1.15)	0.174
**Race**				
Black	Reference			
White	0.93 (0.81~1.06)	0.275	0.88 (0.78~0.99)	0.04
Other	0.73 (0.62~0.87)	< 0.001	0.68 (0.58~0.8)	< 0.001
**Primary site**				
Cardia, NOS	Reference			
Fundus of stomach	1.15 (0.89~1.49)	0.294	1.04 (0.82~1.32)	0.747
Body of stomach	1.07 (0.9~1.27)	0.463	0.97 (0.83~1.14)	0.732
Gastric antrum	1.07 (0.93~1.23)	0.354	1.01 (0.89~1.15)	0.851
Pylorus	1.06 (0.8~1.4)	0.688	1.02 (0.79~1.32)	0.889
Lesser curvature of stomach, NOS	0.92 (0.75~1.12)	0.385	0.88 (0.73~1.05)	0.156
Greater curvature of stomach, NOS	1.02 (0.8~1.3)	0.862	0.96 (0.77~1.2)	0.731
Overlapping lesion of stomach	1.24 (1.04~1.46)	0.014	1.18 (1.01~1.38)	0.041
Stomach, NOS	1.33 (1.14~1.54)	< 0.001	1.24 (1.08~1.43)	0.002
**Summary stage**				
Unknown/unstaged	Reference			
Distant	3.04 (1.85~4.99)	< 0.001	2.46 (1.53~3.93)	< 0.001
Localized	1.42 (0.93~2.17)	0.109	1.4 (0.95~2.06)	0.094
Regional	2.29 (1.52~3.45)	< 0.001	1.95 (1.33~2.85)	0.001
**AJCC stage group**				
Unknown stage	Reference			
IA	0.59 (0.39~0.88)	0.009	0.65 (0.45~0.93)	0.019
IB	0.82 (0.59~1.16)	0.267	0.84 (0.61~1.16)	0.295
II	0.66 (0.46~0.94)	0.021	0.7 (0.5~0.97)	0.034
IIIA	0.73 (0.51~1.05)	0.087	0.78 (0.56~1.09)	0.145
IIIB	1.27 (0.71~2.28)	0.425	1.2 (0.67~2.13)	0.536
IV	0.83 (0.58~1.2)	0.326	0.88 (0.62~1.24)	0.466
**T stage**				
T0	Reference			
T1	0.56 (0.08~4.02)	0.56	0.55 (0.08~3.96)	0.551
T2a	0.62 (0.09~4.48)	0.635	0.62 (0.09~4.49)	0.638
T2b	0.78 (0.11~5.6)	0.805	0.74 (0.1~5.32)	0.765
T2NOS	0.44 (0.06~3.42)	0.43	0.42 (0.06~3.28)	0.412
T3	0.82 (0.11~5.88)	0.842	0.79 (0.11~5.7)	0.818
T4	0.9 (0.13~6.45)	0.915	0.85 (0.12~6.12)	0.875
TX	0.83 (0.11~5.99)	0.852	0.8 (0.11~5.75)	0.82
**N stage**				
N0	Reference			
N1	1.09 (0.96~1.23)	0.173	1.06 (0.95~1.2)	0.307
N2	1.01 (0.83~1.24)	0.896	1.02 (0.84~1.24)	0.866
N3	1.33 (1.04~1.71)	0.025	1.34 (1.05~1.7)	0.017
NX	1.1 (0.9~1.35)	0.363	1.1 (0.91~1.34)	0.333
**M stage**				
M0	Reference			
M1	1.28 (0.92~1.77)	0.139	1.31 (0.95~1.79)	0.097
MX	0.94 (0.69~1.29)	0.716	1.02 (0.77~1.35)	0.892
**Radiation**				
No	Reference			
Yes	0.9 (0.79~1.02)	0.087	0.89 (0.79~1)	0.041
**Chemotherapy**				
No	Reference			
Yes	0.71 (0.63~0.81)	< 0.001	0.71 (0.63~0.8)	< 0.001
**Systemic surgery**				
No	Reference			
Yes	0.75 (0.64~0.87)	< 0.001	0.76 (0.66~0.88)	< 0.001
**Marital**				
No	Reference			
Yes	0.91 (0.81~1.01)	0.089	0.88 (0.8~0.98)	0.017
**Surgery to primary site**				
No	Reference			
Yes	0.35 (0.32~0.38)	<0.001	0.36 (0.33~0.39)	<0.001

**Table 4:** Median survival time of patients according to treatment

	Before PSM		After PSM	
	Surgery vs. non-surgery (HR; 95% CI)	*p* value	Surgery vs. non-surgery (HR; 95% CI)	*p* value
Median OS	24 vs. 4(0.28, 0.27–0.29)	< 0.001	21 vs. 5(0.36, 0.34–0.40)	< 0.001
Median CSS	24 vs. 4(0.27, 0.26–0.29)	< 0.001	21 vs. 5(0.35, 0.32–0.39)	< 0.001

### The Cox regression analysis of the independent prognostic factors for survival in SRCC patients

According to the multivariate Cox analysis, the patients after PSM were independently associated with better CSS (HR = 35%, 95%CI, 0.32–0.38) and OS (HR = 36%, 95%CI, 0.33–0.39). In addition, age, race, summary stage, radiotherapy, chemotherapy, systemic surgery, metastatic sites, resection range, and marital status are all independent factors for the survival of SRCC patients. Specifically, according to Table 3, one of the N stages (N3) was independently correlated with the survival of SRCC patients. Considering that the N stage is an indicator of cancer, the N stage was included in the following multivariate logistic regression and facilitated the establishment of the nomogram.

### A nomogram to determine the best candidate for primary tumor surgery

**Fig. 3:**
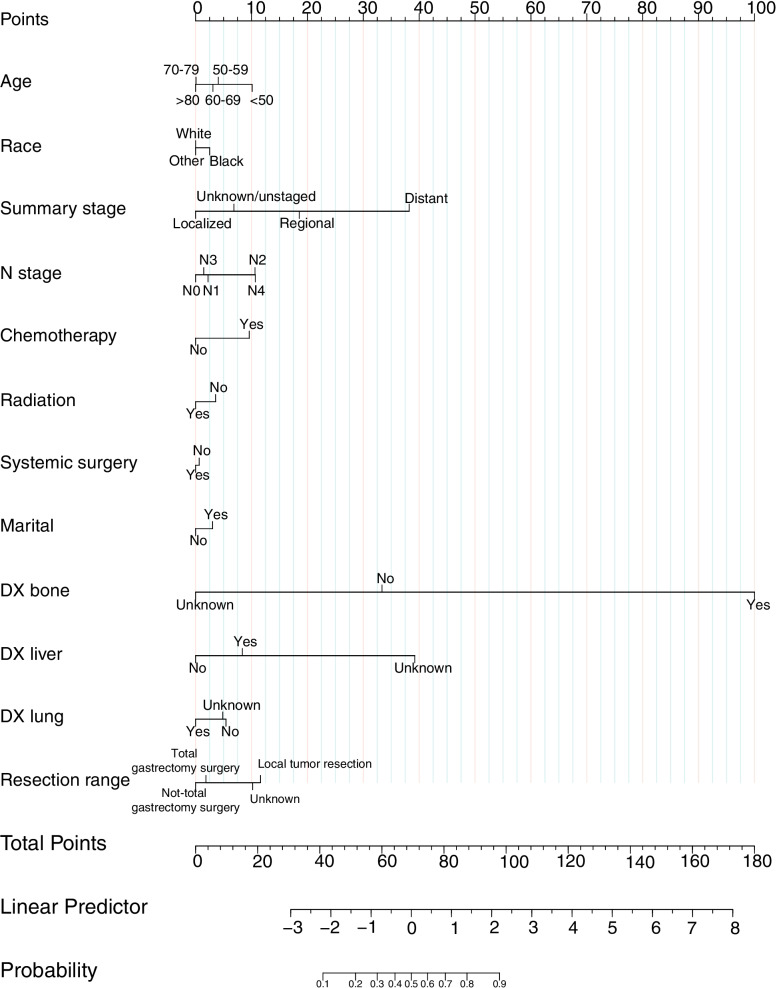
A nomogram to predict optimal candidates for primary tumor resection

The SRCC patients who underwent resection for primary tumors, we assumed, may benefit from surgery if they survive longer than the median CSS time (5 months) of those who did not undergo resection. Among the surgery cohort, 1048 patients (69.8%) survived longer than the median CSS time. Based on the hypothesis, those who underwent resection survived longer than 5 months were placed in the beneficial group and those who lived less than 5 months were placed in the non-beneficial group (Fig. 3).

**Fig. 4:**
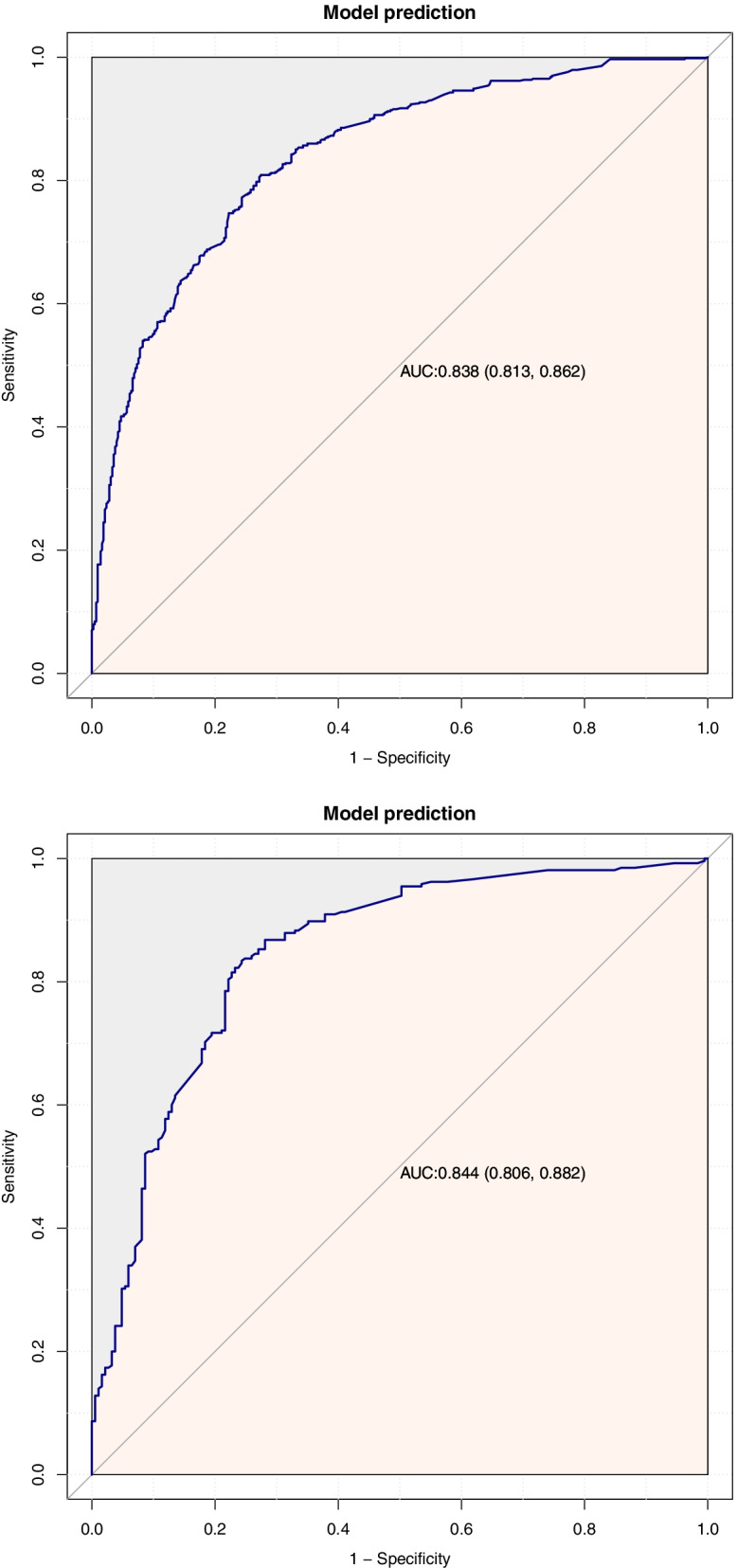
**a** Internal validation. **b** External validation

According to the multivariate Cox analysis of the two groups in the surgery group, the factors that could affect the CSS independently before surgery were included in the training cohort, containing eight indexes: age, race, summary stage, N stage, radiotherapy, chemotherapy, systemic surgery, and marital status. After the multivariate logistic regression, a nomogram was established to forecast the SRCC patients who did benefit from resection for primary tumors in the training set (Fig. 4). The nomogram indicated that the most influential factors were the metastatic sites, summary stage, followed by resection range, N stage, age, chemotherapy, radiation, marital, race, and systematic surgery.

### Verification of the predictive model

**Fig. 5:**
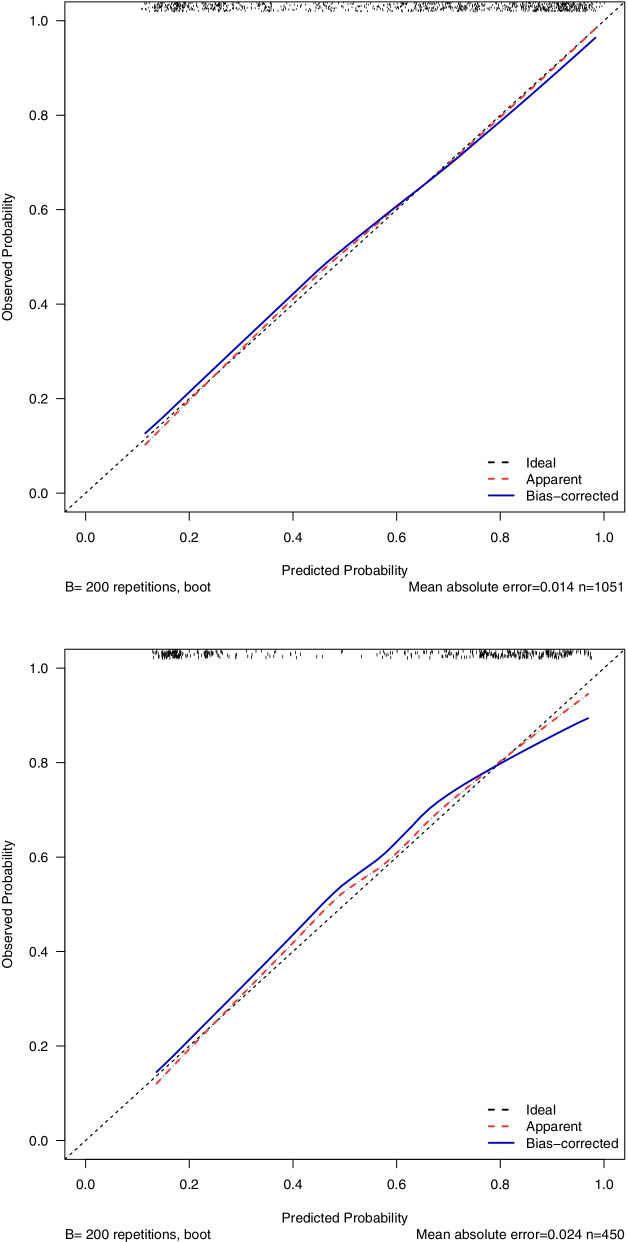
**a** Internal validation. **b** External validation

According to the internal validation of the training set, the AUC index of the nomogram CSS was 83.8% (95%CI, 0.813–0.862) and of the CSS, through external validation with the validation set, was predicted to be 84.4% (95% CI, 0.806–0.882) (Fig. 4). Comparing the internal and external calibration of the OS nomogram, we can know that the prediction of the nomogram is highly correlated with the actual observed results (Fig. 5).

In the verification set, we validated the distinguishability of the model. According to the K-M analysis and the log-rank test, the survival time of the beneficial group was significantly longer than that of the non-beneficial or the non-surgery group.

### Clinical application

The nomogram could be used as a diagnostic tool for clinical research. Applying the nomogram, we first drew a line segment perpendicular to the top row from the corresponding index as the partition point for each factor. Then, we added up the scores at the corresponding points for each factor to arrive at the total score. The probability of the primary tumor resection for SRCC patients can be obtained by making a vertical segment on the line of the total score.

For instance, for a 50-year-old unmarried SRCC patient diagnosed with N3 stage, whose total score would be 35, the nomogram would indicate the probability of the patient benefitting from surgery would be 30%.

## Discussion

From the current researches on SRCC, it is known that a variety of prognostic factors can affect the survival rate of SRCC patients: age, tumor size, tumor stage, radiotherapy, chemotherapy, and surgical resection [9, 10]. Among these factors, surgical resection is the most effective treatment for SRCC patients [8]. However, no research so far has explored and analyzed the clinical characteristics of SRCC patients who are the beneficiaries of surgical resection. Since surgery is potentially risky, there must be some patients who cannot benefit from it. By conducting our research, which established a predictive model capable of better determining the best surgical resection candidates from SRCC patients, we hope that the patients who do undergo surgical resection can benefit from it and that, for those patients who are not suitable, the pain caused by surgery can be avoided.

We derived the analysis data was from the SEER database to construct a nomogram about the survival prognosis of SRCC patients. A new criterion was formulated to build a predictive model capable of further clarifying the candidates who would obtain prolonged survival results from the surgical resection of the primary tumor. The median CSS time of the surgery group was 24 months; that of the non-surgery group was 4. The characteristics of these patients from the surgery and non-surgery groups—age, race, TNM stage, radiation, systemic treatment, and marital status—were integrated into the predictors to create the nomogram. Using the training group for internal validation, the AUC index of the predicted CSS was shown to be 0.846 (95% CI, 0.817–0.875). Using external validation, the AUC of CSS was predicted to be 0.859 (95% CI, 0.817–0.902). These internal and external verifications prove that the constructed nomogram has a certain accuracy.

Due to the selection bias for SRCC patients with favorable individual conditions—smaller tumor, younger age, no distant metastasis—surgical intervention can possibly improve the survival rate. However, surgical resection may have a therapeutic impact that is obscured by selection bias [11]. In our predictive nomogram, chemotherapy and summary stage were the top two strongest predictors of benefit from surgical resection in SRCC patients, demonstrating that certain specific individual conditions are key to choosing different SRCC patients for surgical treatment. Further, younger age, radiation, prior chemotherapy, and systemic treatment make surgery more likely to be beneficial. In other studies, a single small tumor and limited metastasis were associated with better results for SRCC patients undergoing primary tumor surgical resection, which might be due to the lower difficulty of the operation and the relatively good physical condition of the patients [12]. And we found that gastric bare area adipose tissues invasion (GBAI) was identified as a predictor of unfavorable prognosis for gastric cancer and was more commonly found in the proximal or linitis plastica of the stomach than in the distal stomach [13]. But we still do not know the relationship between GBAI and signet ring cell carcinoma. Therefore, additional clinical studies are needed to explore this relationship. As is well known, various factors have different effects on the prognosis. For example, patients with a longer expected survival period and better individual conditions may receive more profitable treatment, such as surgical intervention. Therefore, identifying the specific SRCC patients with potential surgical benefits and performing primary tumor surgery for them may facilitate better treatment. However, in previous studies, no clear selection criteria were found to determine the SRCC patients who would really benefit from surgical resection. The integration of multiple predictors might have a stronger predictive effect than a simple single prognostic indicator [11]. This is why an individualized prediction nomogram was determined as the ideal auxiliary selection tool. Accordingly, the current exploratory research developed an individualized predictive model to determine the real beneficial candidates from the surgical group. Our research, we hope, can offer more predictive information for clinical medicine to make future treatment decisions.

However, according to the data in Table 3, among the TNM stages of tumor metastasis, only the N3 stage was an independent factor affecting the survival rate of patients with SRCC. However, in a study of the clinical features and prognosis of SRCC, the N stage was also an important factor in predicting survival. This is why we included the N stage in the survival analysis to construct the nomogram [14].

Some authors reported that the incidence of signet ring cell carcinoma in Borrmann type IV GC was relatively high [15–17]. Due to the poor prognosis of signet ring cell carcinoma and low sensitivity to chemotherapy, it was suggested that adjuvant chemotherapy might not have a good result, so multimodal therapy was worth further exploring.

Our study only separately considered the effects of radiotherapy and chemotherapy on survival and prognosis; it did not consider radiotherapy and chemotherapy together with surgical resection. Other studies have shown that preoperative radiotherapy was identified as an independent protective factor for a good prognosis of gastric signet ring cell carcinoma [18], suggesting that preoperative radiotherapy could be considered when formulating a treatment strategy for the surgical resection of SRCC [19]. However, one problem still needs to be pointed out: this study may only be suitable for SRCC populations with certain clinical characteristics, so the survival benefits of preoperative radiotherapy may require further exploration.

A study from China has shown that *Helicobacter pylori* eradication therapy can prevent postoperative recurrence of early gastric cancer [20], thus prolonging the overall survival of patients with early gastric cancer. However, the effect of *H. pylori* on primary surgery for signet ring cell carcinoma was not included in our data. More discussions are needed in this area.

In addition, the proportion of patients with distant metastasis and lymphatic invasion in early-onset SRCC was larger than that in late-onset SRCC [21]. Previous studies have not yet fully clarified the pathogenesis of early-onset SRCC patients who are more likely to develop distant metastasis. Some researchers have tried to elucidate the pathogenesis at the molecular biology and genetic level, revealing that early-onset SRCC disease is related to the de novo deletion of CDH1, and CDH 1 is responsible for encoding a protein that plays a role in adhesion junctions. The lack of CDH1 will lead to a decrease in the number of adhesion proteins, thereby making cancer more prone to distant metastasis. The current studies have different opinions on the pathogenesis of early-onset SRCC patients more likely to develop distant metastases, and more exploration should be sought in the future. It is worth noting that although the distant metastasis of patients with early-onset SRCC is more common than that of patients with late-onset SRCC, the survival prognosis of the latter tends to be worse in comparison, which may be due to the poor health status of elderly patients and some complications with poor prognosis [22]. Therefore, to avoid the incomplete dissection of positive lymph nodes, membrane anatomy-guided laparoscopic spleen-preserving circumferential splenic hilar lymph node dissection for advanced proximal gastric cancer is safe and feasible [23]. Some researchers have reported that female sex is associated with lymph node metastasis in early gastric cancer [24–26]. But no studies have shown such a relationship. Further studies were needed to investigate the biological association between sex and lymph node metastasis.

Signet ring cell carcinoma of the stomach is a special type of gastric adenocarcinoma, which is defined as a poorly cohesive carcinoma, with a cell rich in intracytoplasmic mucin pushing the nucleus to the periphery. The difference is that signet ring cell carcinoma of the stomach is known to be more aggressive and invasive than gastric adenocarcinoma [27]. In this case, for signet ring cell carcinoma of the stomach and gastric adenocarcinoma, the choice of therapeutic method will be different. For localized gastric adenocarcinoma, early surgical resection of the local tumor is the only effective treatment [28]. However, other studies have shown that chemotherapy or chemoradiotherapy significantly improves survival compared with surgery alone [29]. In contrast, surgical resection is the most effective treatment for SRCC patients. Moreover, other studies have shown that neoadjuvant chemotherapy has no benefit for survival in advanced SRCC patients [30, 31]. In general, the specific effective treatment for SRCC is not clear enough, so palliative treatment is generally carried out by surgical resection, which is the purpose of our research: we hope that the patients who do undergo surgical resection can benefit from it, and that, for those patients who are not suitable, the pain caused by surgery can be avoided. Additionally, because SRCC is highly invasive, it may be transferred to the peritoneum, forming peritoneal surface malignancies. Some authors believe that cytoreductive surgery and hyperthermic intraperitoneal chemotherapy should be a powerful means to treat this kind of surface malignant tumors [32].

This study has some limitations. First, it is a systematic retrospective analysis. Like any observational study, there is an inherent recall bias that cannot be eliminated. Meanwhile, the SEER database lacks information on performance status and comorbidities that may lead to selection bias in therapy choices. Second, due to the lack of clinical information and data for patients with advanced SRCC (stage II–III) in the SEER database, we failed to conduct a comprehensive analysis of them. As some studies had shown, adjuvant CRT could improve the survival and prognosis of patients with SRCC stage II–III [33]. At the same time, the SEER database does not accurately classify surgical methods (radical/palliative surgery). Therefore, this study cannot conduct an in-depth and strict discussion on these two factors. In this study, the median OS of SRCC patients in the adjuvant radiotherapy group was significantly longer than that in the surgical resection group, revealing that for patients with stage II–III SRCC, surgical resection of the primary tumor may not be the best choice. In the future, other treatment modes, such as multiple treatment modes, are worthy of further exploration. In addition, the experimental method of this study can also be improved. First, the majority of studies were performed in the Americas. The differences in dietary habits and environmental deviation may affect the results of the survey. Second, this meta-analysis did not involve RCT evaluation, and our studies were limited to retrospective studies, which might pose a potential risk of bias risks. Third, we calculated the HR estimates from the Kaplan-Meier survival curves, which potentially reduces the reliability of the results. Finally, in the process, the statistical heterogeneity was high, which led to inexorable biases in the result. In short, we found that additional data plays a critical role in producing the meta-analysis of improved quality and reliability.

## Conclusion

Based on the above analysis, we put forward a model to identify patients with SRCC who would benefit from surgery. We can learn from our predictive model that among SRCC patients, younger age, localized (tumor having not metastasized), N0, radiation and chemotherapy, not systemic surgery, and being married are all likely to benefit more from surgery. At the same time, we established a model to identify the patients who will not benefit from surgery. Although surgery is the first choice according to the current clinical guidelines, these patients can choose other better treatment methods, which is particularly important.

## Data Availability

SEER belongs to public databases. The patients involved in the database have obtained ethical approval. Users can download relevant data for free for research and publish relevant articles. Our study is based on open-source data, so there are no ethical issues and other conflicts of interest.
